# Circadian and homeostatic modulation of functional connectivity and regional cerebral blood flow in humans under normal entrained conditions

**DOI:** 10.1038/jcbfm.2014.109

**Published:** 2014-06-18

**Authors:** Duncan J Hodkinson, Owen O'Daly, Patricia A Zunszain, Carmine M Pariante, Vitaly Lazurenko, Fernando O Zelaya, Matthew A Howard, Steven C R Williams

**Affiliations:** 1 Department of Neuroimaging Sciences, Institute of Psychiatry, King's College London, London, UK; 2 Laboratory of Stress, Psychiatry and Immunology, Department of Psychological Medicine, Institute of Psychiatry, King's College London, London, UK

**Keywords:** CBF, circadian, connectivity, cortisol, diurnal, homeostatic

## Abstract

Diurnal rhythms have been observed in human behaviors as diverse as sleep, olfaction, and learning. Despite its potential impact, time of day is rarely considered when brain responses are studied by neuroimaging techniques. To address this issue, we explicitly examined the effects of circadian and homeostatic regulation on functional connectivity (FC) and regional cerebral blood flow (rCBF) in healthy human volunteers, using whole-brain resting-state functional magnetic resonance imaging (rs-fMRI) and arterial spin labeling (ASL). In common with many circadian studies, we collected salivary cortisol to represent the normal circadian activity and functioning of the hypothalamic–pituitary–adrenal (HPA) axis. Intriguingly, the changes in FC and rCBF we observed indicated fundamental decreases in the functional integration of the default mode network (DMN) moving from morning to afternoon. Within the anterior cingulate cortex (ACC), our results indicate that morning cortisol levels are negatively correlated with rCBF. We hypothesize that the homeostatic mechanisms of the HPA axis has a role in modulating the functional integrity of the DMN (specifically, the ACC), and for the purposes of using fMRI as a tool to measure changes in disease processes or in response to treatment, we demonstrate that time of the day is important when interpreting resting-state data.

## Introduction

By nature, humans are diurnal creatures. Our physiology and behavior are driven by an endogenous oscillator with a period of ∼1 day, hence the term circadian rhythm. Under normal conditions, these circadian rhythms are synchronized (or entrained) to the external environment, principally via light–dark cycles^1^ but also via social routines.^2^ The importance of entrained clocks is that they enable us to anticipate and thereby prepare for the challenges and opportunities of day and night and adapt to seasonal changes. Disturbances of these temporal relationships will inevitably lead to circadian variation in health and disease.^3^

In humans, the most overt manifestation of circadian rhythmicity is the sleep–wake cycle. The timing of sleep is dictated by two interacting processes: the circadian component, which arises from the suprachiasmatic nuclei and drives a nearly 24-hour endogenous oscillatory process,^4^ and the homeostatic component, which arises from the preoptic area and increases with time spent awake.^5, 6^ Both these systems appear to be highly interconnected anatomically and functionally,^7^ with evidence to suggest that circadian genes might affect sleep (the hallmark of the homeostatic process) and cognition.^8^ Indeed, many higher-order cognitive processes, such as attention, memory, and executive function appear to vary with the time of day.^9^ It may therefore be the case that circadian clocks optimize our daily performance by facilitating widespread changes in brain activity and plasticity.^10^

Neuroimaging studies of diurnal variation are rare, but the data available suggest that neuronal activity of the brain at rest may fluctuate over the course of a single day. This has been observed as daily changes in cortical activity using low-resolution electromagnetic tomography,^11^ and as differences in regional brain glucose metabolism using [18F]-fluorodeoxyglucose positron emission tomography.^12^ Daily fluctuations in spontaneous brain activity have also been observed with resting-state functional magnetic resonance imaging (rs-fMRI),^13, 14^ indicating reduced connectivity strength and spatial extent within the default mode network (DMN) as the day progresses.^13^ However, it is still unclear whether these daily changes in functional connectivity (FC) and their respective energy demands are regulated by the homeostatic mechanisms of the neuroendocrine system.

The aim of the present study was to investigate the effects of circadian and homeostatic processes on FC and regional cerebral blood flow (rCBF) in healthy human volunteers, using whole-brain rs-fMRI and arterial spin labeling (ASL). Arterial spin labeling is primarily insensitive to low-frequency noise;^15^ therefore, it is the ideal modality to detect blood flow changes taking place on much slower timescales. We also wished to consider the basal adrenocortical activity of each subject to ensure the same physical dimension over time. In common with many circadian studies, we used salivary cortisol levels to represent the normal circadian activity and identify potential stress responses or disturbances in the functioning of the hypothalamic–pituitary–adrenal (HPA) axis. Under normal entrained conditions, we hypothesize that daily changes of functional integration within the DMN are influenced by underlying changes in the HPA axis.

## Materials and Methods

### Subjects

Thirteen right-handed, healthy adult male volunteers were recruited for the study (mean age (±s.d.) 27±4; range 23 to 38 years, mean weight 80±12 kg). Exclusion criteria included history of brain injuries, hypertension, any psychiatric or neurologic disease, alcohol or drug abuse, and symptoms of sleep problems such as insomnia, obstructive sleep apnea, narcolepsy, or restless legs syndrome. Any volunteers that were acutely ill, with fever and malaise, were excluded or rescheduled after full recovery.

The study was approved by King's College Hospital Research Ethics Committee (REC reference 04/Q0706/72). Informed, written consent was provided by all participants.

### Daily Activities and Lifestyle Restrictions

For the duration of the study, subjects maintained a typical sleep schedule. They were asked not to alter their current exercise regimen or the types of activities that they would normally perform. Consumption of high-protein meals was also avoided, as it is known to cause cortisol secretory episodes.

### Study Design

Participants were scanned on two separate days: once in the morning (between 0800 and 1000 hours) and once in the afternoon (between 1500 and 1900 hours). The order of morning and afternoon visits was randomized to account for scan order effects. In addition to the imaging outcomes, we investigated the basal HPA axis function from saliva cortisol samples. On the day of each MRI examination (i.e., morning and afternoon scans), saliva samples were collected at three fixed time points (1200, 0400, and 0800 hours), along with measurements of the cortisol awakening response (CAR) (i.e., at awakening (0 minutes), 30 minutes after waking, and 60 minutes after waking).^16, 17^ Two additional samples were collected immediately before and after scanning to monitor the subjects stress response to the imaging procedures.

### Salivary Cortisol

Cortisol measurements were obtained according to a previously established protocol.^18^ Participants used polymer swabs (Salimetrics Oral Swabs, Salimetrics, PA, USA) to collect saliva, which were then inserted into Salivette tubes (Sarstedt; Leicester, UK). Instructions to participants were not to have breakfast or brush their teeth until after the final waking sample had been collected. For samples taken throughout the day, participants were asked not to eat or drink 30 minutes before collecting the saliva. Samples were kept in the fridge until the volunteers were able to bring them to the laboratory, at which point, they were frozen at −20 °C until ready to be analyzed.

Saliva cortisol concentration was measured with a commercial immunoassay kit (High Sensitivity Salivary Cortisol ELISA KIT from Salimetrics) after the recommended procedure. Optical density was read at 450 nm with correction at 620 nm, using a Beckman Coulter (Brea, CA, USA) DTX 880 plate reader, with Multimode Detection Software 2.0.0.12. All samples from the same subject were analyzed in the same run. The data were analyzed as previously described.^18^

### Magnetic Resonance Imaging Acquisition

Participants were scanned in the supine position on a 3T whole-body MRI scanner (GE, Milwaukee, WI, USA) using an 8-channel receive-only phased array head coil. For image registration, a high-resolution T2-weighted anatomic image was acquired at both morning and afternoon visits (fast spin echo, slice thickness=2 mm, field of view=240 mm^2^, matrix=320 × 320, repetition time/echo time=4380/55, in-plane resolution=1.875 × 1.875 mm^2^). Resting-state functional magnetic resonance imaging based on the blood–oxygen level-dependent signal was acquired using a T2*-weighted sequence (gradient echo echo planar imaging, 37 slices, 2.4 mm slice thickness, 1 mm slice gap, in-plane resolution=3.4 × 3.4 mm^2^, field of view=218 mm^2^, matrix=64 × 64, repetition time/echo time=2,000/30 ms, FA=75°, 256 volumes). Cerebral perfusion was measured using a pseudo-continuous ASL sequence^19^ (three-dimensional fast spin echo interleaved spiral readout, 8 shots, TE/TR=32/5,500 ms, ETL=64, 3 tag-control pairs, labeling duration=1.5 seconds, post-labeling delay=1.5 seconds, spatial resolution=1 × 1 × 3 mm). Full details of the pseudo-continuous ASL sequence and absolute quantification of CBF using a single-proton density image are discussed previously.^20^ Throughout the imaging procedure, participants were instructed to lie still with their eyes open.

### Preprocessing

All imaging data were processed using statistical parametric mapping (SPM) 8 (http://www.fil.ion.ucl.ac.uk/spm). To ensure both imaging modalities were consistently aligned, we acquired high-resolution T2-weighted structural images at both the morning and afternoon visits. Both the ASL and echo planar imaging scans were co-registered independently using rigid body transformation to the same structural scan for each session, before performing spatial normalization. The spatial normalization parameters required to warp the T2 image to Montreal neurological institute (MNI) standard space were estimated (via SPM unified segmentation) and these transformation parameters were applied to all functional images. Both data sets were spatially smoothed using a 5 mm FWHM (full-width at half-maximum) Gaussian kernel to accommodate for gyral variability across subjects.

### Functional Connectivity Analysis

Preliminary checks for the rs-fMRI data consisted of slice timing correction and realignment to correct for motion artifacts. To condition the data for voxel-wise correlations, the time series were linear detrended and temporally band-pass filtered (0.01–0.1 Hz). The influences of several confounding signals were removed through linear regression (including six motion parameters, mean white matter time course, and mean cerebrospinal fluid time course). The global signal was not removed from the data, as recent evidence suggests that global signal regression may give rise to artifactual anti-correlated networks.^21^ White matter and cerebrospinal fluid time courses were extracted using thresholded *a priori* tissue probability maps provided in SPM (cerebrospinal fluid >60% and white matter >80%).

For FC, we used seed-based correlation based on six previously identified seed regions.^22^ These regions comprise 12 mm-diameter radius spheres centered on the intraparietal sulcus (−25, −57, 46), the frontal eye field region of the precentral sulcus (25, −13, 50), the middle temporal region (−45, −69, −2), MPF (−1, 47, −4), posterior cingulate and precuneus (PCC; −5, −49, 40), and lateral parietal cortex (−45, −67, 36) (see Supplementary Figure S1). The correlation coefficient (*r*) maps were converted to a normal distribution by Fisher's *z* transform, and the group *z*-score maps for the six seed regions were combined using a conjunction analysis and the method of Minimum Statistic compared with the Global Null.^23^ These conjunction analyses retain voxels with main effects of condition but a ‘null' interaction effect between them. Session-wise changes in FC were calculated under the framework of the general linear model using a random effects flexible factorial analysis of variance model.

### Cerebral Blood Flow Analysis

Quantification of the ASL data was performed in native space as described previously.^19, 20^ All CBF maps were preprocessed as described in Preprocessing section. Gray matter masking was applied in standard MNI space using the SPM probabilistic image thresholded at 20% and binarized to create an exclusive mask. This was primarily done because of the well-established lack of sensitivity of ASL in white matter. To determine the session-wise changes in regional CBF, a random effects second-level analysis was performed using a paired *t*-test.

### Correlation Analysis

Voxel-wise analysis was performed to identify brain regions that show a significant correlation between CBF/FC values and the cortisol data. The SPM multiple regression analysis is an implementation of the general linear model using the theory of Gaussian random fields. Hormone levels were entered as a covariate of interest in this model, and only results significant after correction for multiple comparisons were reported. To prevent biasing, the individual subjects CBF/FC values were extracted from anatomically derived regions of interest using the automated anatomical labeling atlas.^24^

## Results

### Cortisol

**Figure 1:**
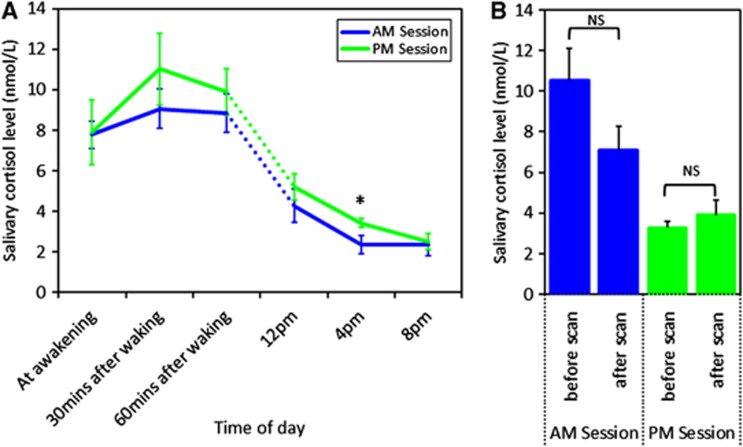
Cortisol measurements. (**A**) Cortisol awakening response and diurnal cortisol decline on the day of the magnetic resonance imaging examinations. (**B**) Cortisol levels immediately before and after scanning. The order of the morning (blue) and afternoon sessions (green) was randomized to account for scan order effects. Data represent the mean±s.e.m. (*P<0.05 after general linear model (GLM); NS, not significant after paired *t*-test).

Figure 1A shows the mean CAR and diurnal cortisol decline obtained from all subjects and sessions. As expected, on both occasions, cortisol secretion was highly rhythmic with declining levels throughout the day, and a sharp rise just after waking. The peak response occurred 30–40 minutes after waking; and by 60 minutes, cortisol levels were beginning to decrease toward the levels at awakening. Summary measures of the cortisol decline and CAR are shown in Table 1. We found a significant difference in the cortisol levels at 0400 hours (general linear model, *P*=0.0497), but the CAR indices were not significantly different between the collection days (paired Student *t*-test). There were no significant differences in the cortisol samples taken immediately before and after the magnetic resonance imaging scans (paired Student *t*-test: AM session *P*=0.057, PM session *P*=0.350). These results are displayed in Figure 1B.

### Session-Wise Changes in Functional Connectivity

**Figure 2:**
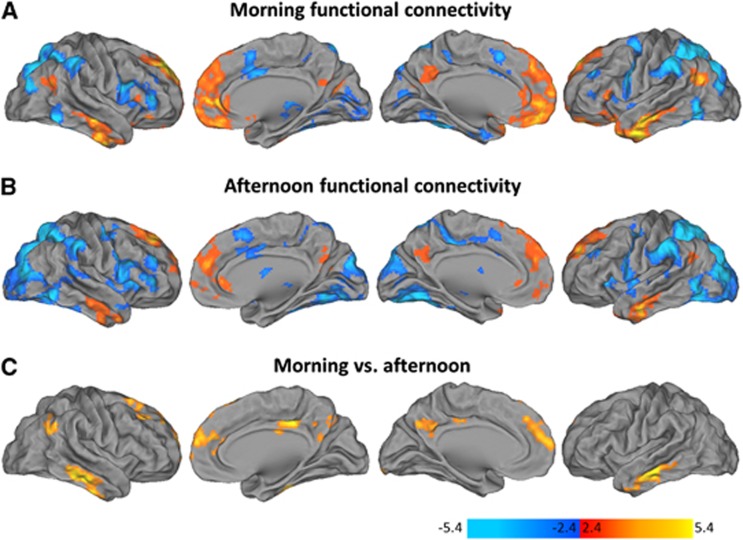
Daily variations in functional connectivity (FC). The maps illustrate the conjunction analyses across all seeds in the morning (**A**) and afternoon (**B**). Session-wise changes in FC are displayed in the bottom row (**C**) (contrast: morning (AM)>afternoon (PM)). Task-negative and task-positive nodes are shown in warm and cool colors, respectively. Images are displayed with a cluster probability threshold of *P*<0.05, corrected for multiple comparisons (family-wise error). The seed-based analysis employed six regions as previously described in *Fox et al.*^22^ These are shown in Supplementary information, Supplementary Figure S1.

The DMN of FC was readily detectable in the conjunction maps from both morning and afternoon sessions (Figure 2). Regions exhibiting coherent activity in the DMN included PCC, bilateral inferior parietal lobules, medial prefrontal cortex, and temporal cortex. The task-positive network consisted of regions in the dorsolateral prefrontal cortex, ventrolateral prefrontal cortex, insula, and supplementary motor area. Decreased synchrony was detected in the DMN during the afternoon (i.e., AM>PM). These decreases were predominantly localized to midline association regions and important connector nodes, such as the PCC, medial prefrontal cortex, and temporal cortex. No significant changes were identified in the task-positive network.

### Session-Wise Changes in Cerebral Blood Flow

**Figure 3:**
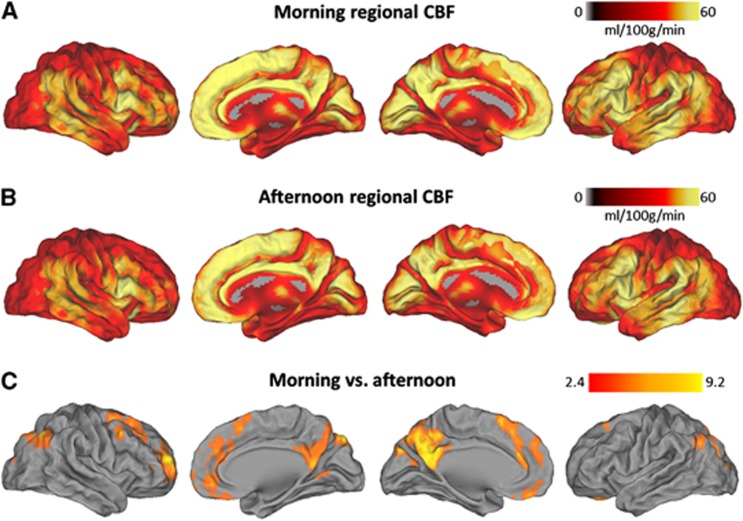
Daily variations in regional cerebral blood flow (CBF). The maps illustrate the average CBF values (ml/100 g/minutes) across all subjects in the morning (**A**) and afternoon (**B**). Session-wise changes in regional CBF are displayed in the bottom row (**C**) (contrast: morning (AM)>afternoon (PM)). Images are displayed with a cluster probability threshold of *P*<0.05, corrected for multiple comparisons (family-wise error).

In the afternoon, the group-level results (Figure 3) revealed significant CBF decreases in the core regions associated with the default network, including the PCC, bilateral inferior parietal lobules (including angular gyrus and middle temporal gyrus), and medial prefrontal cortex (including medial and middle frontal gyrus, and ACC). No significant regions of increased perfusion were observed in response to the time of the day. Furthermore, there were no significant differences in global grey matter CBF values between the morning and afternoon sessions (paired Student *t*-test: *P*=0.654).

### Correlation between Functional Connectivity and Regional Cerebra Blood Flow

**Figure 4:**
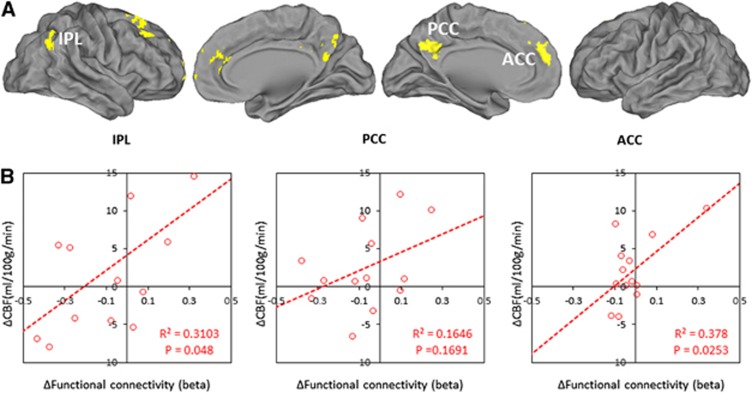
(**A**) Common patterns of daily variations in functional connectivity (FC) and regional cerebral blood flow. Areas of overlap between both modalities are displayed in yellow. (**B**) Correlations between the change in (Δ) FC and ΔCBF within the three anatomic areas. ACC, anterior cingulate cortex; IPL, inferior parietal lobule; PCC, posterior cingulate and precuneus.

Figure 4A shows the common patterns of daily variation in FC and rCBF. The PCC, ACC, and inferior parietal lobule are revealed as regions showing considerable overlap across the maps. For each of these anatomic areas, the linear dependence between rCBF and FC was examined (Pearson's correlation coefficient). Our results show that the mean magnitude of the rCBF decreases correlate positively with the decrease in FC (see Figure 4B). The regions with the strongest relationship were the inferior parietal lobule and ACC. Data from the PCC were not significantly correlated.

### Correlation between Regional Cerebral Blood Flow and Cortisol

**Figure 5:**
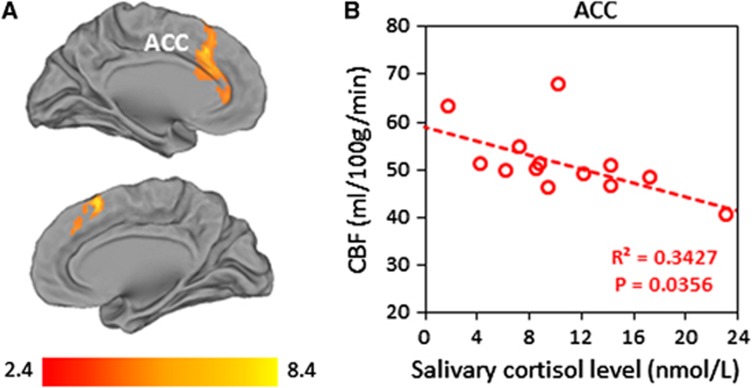
Correlation between regional cerebral blood flow (CBF) and cortisol in the morning. (**A**) Whole-brain voxel-wise regression analysis of regional CBF and cortisol values taken immediately before scanning. Areas are displayed with a cluster probability threshold of *P*<0.05, corrected for multiple comparisons (family-wise error). (**B**) Negative correlation between each subject's mean CBF values and salivary cortisol levels in the anterior cingulate cortex (ACC).

At the morning visits only, we found a significant negative correlation between rCBF and cortisol in the ACC (Figure 5A). This relationship is illustrated with the scatter plot in Figure 5B, which shows the correlation between each subjects mean CBF values in the ACC with the cortisol values taken immediately before scanning.

## Discussion

### Basal Hypothalamic–Pituitary–Adrenal Axis Function

The CAR and diurnal cortisol decline have been used as indexes of HPA axis function in various research fields. Cortisol awakening response and cortisol decline represent two separate adrenocortical functions: CAR signifies the activation (or reactive capacity) of the HPA axis after awakening,^17^ and the cortisol decline represents adrenal secretory activity over the remainder of the diurnal cycle.^25^ Our results show that the CAR is highly stable within the subjects from session to session (see Table 1). In the case of the diurnal cortisol decline, we found a significant difference in the cortisol levels at 0400 hours, but the remaining diurnal profile was not significantly different between collection days. Interestingly, there was a trend for lower cortisol after scanning during the morning sessions (AM session *P*=0.057); however, we do not attribute this to stress because cortisol naturally drops more rapidly in the morning. Overall, the patterns of hormone secretion were analogous to those reported previously in healthy humans.^26^ Our findings confirm that participants had no unforeseen stressful events during the study, and were otherwise healthy across visits. Furthermore, the subjects' adrenocortical activity was within the normal homeostatic range, and they were scanned in a stable allostatic state at both visits.^27^

It is important to remember that salivary cortisol is a peripheral measure and that secretory patterns may deviate without necessarily reflecting dysregulation at a more central level. Salivary cortisol sampling has become increasingly common over the past few years, and it is well documented that the level of cortisol in saliva represents the free form of the steroid hormone in blood serum. An added benefit of collecting saliva is that it is much easier than venepuncture and can be readily repeated at frequent intervals. Furthermore, this approach is free from the stress or novelty of hospital settings as well as the trouble and expense of bringing healthy subjects to the clinic; hence, we believe ambulatory procedures have some distinct advantages.

### Daily Variations in Functional Connectivity

The anatomy of the default network has been characterized using multiple approaches.^28^ To investigate the daily variations in FC, we measured the interregional temporal correlations in blood–oxygen level-dependent signal fluctuations. Much like other resting-state functional magnetic resonance imaging studies, we revealed a highly correlated, DMN and task-positive network that were similar in the morning and afternoon.^22^ Within these patterns of synchronous activity, significant decreases in correlation strength and spatial extent were observed in the afternoon between major nodes of the DMN. Studies of this nature are rare, but our results are in many aspects consistent with the work of Blautzik *et al*,^13^ who demonstrated daily oscillating patterns in the FC of two DMN subsystems, each peaking early in the morning and declining thereafter. However, it is important to note that there are potential differences owing to the variations in the methodology and study design, and particularly with reference to individual internal time (i.e., chronotype). Here we advocate that under normal (entrained) conditions, FC is altered with the time of day, and this reflects a possible adaptive change in functional integration. Confirmatory results were found when we analyzed the resting-state ASL data.

### Daily Variations in Regional Cerebral Blood Flow

Human positron emission tomography studies of circadian variation and interaction with sleep have shown varying effects on brain metabolism.^29, 30, 31, 32, 33^ These conflicting results may depend on the different imaging strategies used to measure brain metabolism, the conditioning of subjects, and the fact that these studies tended only to apply to sleep; hence, the effects might therefore not be directly comparable. Here we used ASL to investigate the effects of diurnal variation on whole-brain CBF. As it is primarily insensitive to low-frequency noise,^15^ it can be used to quantify physiological changes taking place on much slower timescales. These advantages allowed us to identify regional decreases in CBF moving from the morning to the afternoon. This occurred within a set of brain regions remarkably similar to the DMN, including midline association regions and lateral parietal cortices. Regional CBF (rCBF) is closely coupled with glucose utilization, oxygen consumption, and aerobic glycolysis in the resting human brain.^34, 35^ A tight coupling between blood flow and brain functional topology has also been demonstrated during rest and in response to task demands.^36^ We therefore speculate that the decreased perfusion within the important connector nodes is potentially reflecting daily fluctuations of activity in the DMN.

### Coupling Between Functional Connectivity and Regional Cerebral Blood Flow

A major new finding of our study is the fact that FC changes were closely correlated with the changes in rCBF (see Figure 4). There is strong evidence to suggest that resting-state blood–oxygen level-dependent functional magnetic resonance imaging-derived measures are tightly coupled with rCBF and that these are linked to regional spontaneous brain activity.^37, 38^ Under the widely accepted premise that synaptic strength depends on synaptic activity, this would be consistent with the hypothesis that there is a reorganization of synchronized network activity to a configuration that is less energy demanding in the afternoon, but at the cost of integration capacity. It has been hypothesized that the default network supports a broad low-level focus of attention when one passively monitors the external world for unexpected events.^28^ In this context, a loss of synchrony in the network might reflect a natural decline in our ability to support exploratory monitoring of the external environment, and thus our readiness to respond to sensory stimuli. As the majority of human biologic functions show daily fluctuations, it is also conceivable that the central processing of complex multimodal functions may also be rhythmic. Hence, diurnal variations of clinical relevance should be detectable despite differences in methods.

### Cerebral Blood Flow Association with Morning Cortisol

Within the ACC, our results demonstrate that morning cortisol levels are negatively correlated with regional CBF (see Figure 5). The reasons for this relationship are unclear, but could be related to the actions of cortisol on glucocorticoid and mineralocorticoid receptors. These receptors are known to be expressed in the hippocampus, prefrontal cortex, amygdala, and hypothalamus.^39^ Animal lesion studies suggest that the negative feedback processes of glucocorticoids are partially communicated via the medial prefrontal cortex, including the cingulate gyrus.^40^ Given the overlapping anatomy of the imaging findings and the brain regions involved in feedback control, we speculate that the homeostatic mechanisms of the HPA axis have a key role in modulating the functional integrity of the DMN, and specifically the ACC appears to exert a strong circadian bias in the morning (see Figure 5). The importance of the ACC in behavioral, emotional, and autonomic responses to stress has been well established.^41^ However, it is important to remember that the HPA axis is a complex and dynamic system, and salivary cortisol measures can provide only a partial window into how this system is regulated. Direct evidence for synaptic remodeling in the ACC will need to be confirmed from more invasive experiments.

## Conclusion

Whatever the underlying mechanisms of the present findings, especially for the purposes of using functional magnetic resonance imaging as a tool to measure changes in disease processes or in response to treatment, we demonstrate that circadian and homeostatic influences are important when interpreting the results of resting-state data. Our findings can be of great value for the interpretation of published results, particularly those reporting changes in DMN activity and functions of the ACC. This may also have significant repercussions both for clinical neuroimaging and for the interpretation of the experimental results gathered in patient populations (i.e., patients with neuroendocrine and metabolic disturbances). One recommendation when designing future imaging studies (cross-over and independent groups) is to carefully control for the time of day effects. This is especially important in the morning, when hemodynamic parameters (i.e., regional CBF) appear to carry a strong circadian bias.

## Figures and Tables

**Table 1:** Summary statistics of the daily cortisol decline and awakening response

*Characteristics*	*Morning session*		*Afternoon session*		P *value*
	*Mean*	*s.e.m.*	*Mean*	*s.e.m.*	
*Cortisol measurements*					
At awakening	7.775	(1.595)	7.827	(0.783)	0.295
30 minutes after waking	9.054	(1.792)	11.552	(1.058)	0.176
60 minutes after waking	8.859	(1.145)	10.577	(0.946)	0.939
01200 hours	4.272	(0.655)	5.476	(0.939)	0.923
1600 hours	2.352	(0.231)	3.643	(0.516)	0.049*
2000 hours	2.362	(0.389)	2.355	(0.642)	0.544
					
*CAR-derived parameters*					
AVE	8.562	(1.386)	9.985	(0.718)	0.348
AUC_B_	466.477	(95.681)	469.615	(46.997)	0.981
AUC_G_	521.106	(88.377)	622.617	(47.566)	0.282
AUC_I_	54.629	(38.670)	153.001	(50.804)	0.073
PK	11.038	(1.583)	12.331	(0.935)	0.391
RT	1.084	(1.197)	2.750	(1.244)	0.323
SP	0.184	(0.026)	0.206	(0.016)	0.391
TBP	39.231	(7.113)	36.923	(4.985)	0.794
SBP	0.072	(0.017)	0.124	(0.034)	0.135

AUC_B_, area under the curve about the baseline; AUC_G_, area under the curve about the ground; AUC_I_, area under the curve above the baseline; AVE, average of the three waking cortisol measurements; CAR, cortisol awakening response; GLM, general linear model; PK, peak cortisol; RT, reactivity (the change in salivary cortisol during the awakening period); SBP, slope from baseline to peak; SP, slope of the line through the baseline and last measuremt (60 minutes after waking); TBP, time in minutes from baseline to peak.

Data are reported as the mean±s.e.m. *P* values are calculated using GLM or Paired *t*-test; *P<0.05.
