# Supplementary material for: Circadian and homeostatic modulation of functional connectivity and regional cerebral blood flow in humans under normal entrained conditions
Source: J Cereb Blood Flow Metab. 2014 Jun 18;34(9):1493–9. doi: 10.1038/jcbfm.2014.109 (PMC4158665; doi:10.1038/jcbfm.2014.109)
Supplement: Supplementary Figure S1 [file jcbfm2014109x1.doc]

# Supplimentary INformation

**
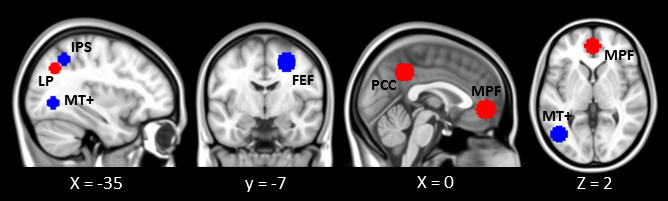
**

**Figure S1:** Six a priori seed regions within the default network (red) and task-positive network (blue). [Abbreviations: IPS, intraparietal sulcus; FEF, frontal eye field (FEF) region of the precentral sulcus; MT+, middle temporal region; MPF, medial prefrontal; PCC, posterior cingulate-precuneus; LP, lateral parietal cortex.]
